# Multi‐Shank 1024 Channels Active SiNAPS Probe for Large Multi‐Regional Topographical Electrophysiological Mapping of Neural Dynamics

**DOI:** 10.1002/advs.202416239

**Published:** 2025-02-27

**Authors:** Gian Nicola Angotzi, Mihály Vöröslakos, Nikolas Perentos, Joao Filipe Ribeiro, Matteo Vincenzi, Fabio Boi, Aziliz Lecomte, Gabor Orban, Andreas Genewsky, Gerrit Schwesig, Deren Aykan, György Buzsáki, Anton Sirota, Luca Berdondini

**Affiliations:** ^1^ Fondazione Istituto Italiano di Tecnologia Microtechnology for Neuroelectronics Unit (NetS3 lab) via Morego 30 Genova 16163 Italy; ^2^ Corticale Srl via Pietro Chiesa 9 Genova 16149 Italy; ^3^ Neuroscience Institute Grossman School of Medicine New York University 550 First Avenue New York NY 10016 USA; ^4^ Faculty of Biology Ludwig‐Maximilians‐Universität Großhaderner Straße 2 82152 Munchen Germany; ^5^ University of Nicosia School of Veterinary Medicine 21 Ilia Papakyriakou 2414 Nicosia Cyprus; ^6^ Department of Neurology Grossman School of Medicine New York University 435 East 30th Street New York NY 10016 USA

**Keywords:** hippocampal and cortical networks, large‐scale electrophysiology, neural dynamics, neural interfaces, neural probes

## Abstract

Implantable active dense CMOS neural probes unlock the possibility of spatiotemporally resolving the activity of hundreds of single neurons in multiple brain circuits to investigate brain dynamics. Mapping neural dynamics in brain circuits with anatomical structures spanning several millimeters, however, remains challenging. Here, a CMOS neural probe advancing lateral sampling for mapping intracortical neural dynamics (both LFPs and spikes) in awake, behaving mice from an area >4 mm^2^ is demonstrated. By taking advantage of SiNAPS technology modularity, an 8‐shank probe with 1024 recording channels arranged in regular arrays of 128 electrodes/shank with an electrode pitch <30 µm is realized. Continuous low‐noise recordings (spikes with 6.67 ± 1.02 µV_RMS_) from all 1024 electrodes at 20 kHz/channel demonstrate the monitoring at high spatial and temporal resolution of a field of view spanning the 2D lattice of the entire mice hippocampal circuit, together with cortical and thalamic regions. This arrangement allows combining large population unit monitoring across distributed networks with precise intra‐ and interlaminar/nuclear mapping of the oscillatory dynamics.

## Graphical abstract

This research article introduces a novel CMOS neural probe with 1024 channels distributed across eight shanks. The SiNAPS probe allows for the simultaneous recording of both local field potentials and action potentials in large brain areas. The probe's design allows for detailed mapping and linking neural dynamics and population unit activity, as illustrated in the hippocampus of mice, revealing new insights into the relationship between neural activity and brain structure.

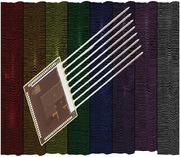

## 1 Introduction

Combining the two facets of single neuronal population activities and oscillatory dynamics in multichannel extracellular recordings requires high‐resolution, large‐scale sensing devices capable of monitoring spiking and local field potentials (LFP) within and across widely distributed brain circuits.^[^ ^1^, ^2^, ^3^ ^]^ These competing needs can be achieved by radical scaling of the number of recorded electrodes to yield dense sampling across circuits. Recent studies^[^ ^4^, ^5^, ^6^, ^7^ ^]^ demonstrated micro‐/nano fabricated implantable Complementary Metal‐Oxide Semiconductor (CMOS) probes with hundreds to thousands of recording electrode‐pixels within small cross‐sectional areas. By integrating into the same silicon substrate electrodes and electronic circuits for signal amplification, filtering, and read‐out, such implantable CMOS probes can simultaneously record from multiple brain regions^[^ ^4^, ^8^ ^]^ with an unprecedented spatial resolution. Furthermore, the high 2D spatial resolution of these probes also permits the use of advanced automated sorting algorithms to better isolate putative single‐unit activity, by exploiting correlations of individual neuron spikes measured by closely and regularly spaced electrode contacts.^[^ ^8^, ^9^, ^10^ ^]^ Yet, continuously monitoring a large field of view at the highest probe spatial resolution with current CMOS‐based probe implementations is limited either by the unequal number of available electrodes and output channels or by the probe layout. Although the tremendous success achieved in recordings with Neuropixel probes, their channels‐to‐electrodes ratio < 1 (i.e., 384/960 = 0.4 in Neuropixel 1.0^[^ ^11^ ^]^; 384/5120 = 0.075 in Neuropixel 2.0^[^ ^12^ ^]^; 1536/5120 = 0.3 in Yang, et. al., 2024^11^) restricts the continuous spatial coverage at the highest electrode density, thus imposing to the user a trade‐off between the spatial coverage and the recording channel density based on the available front‐end channels. Further, available layouts are either limited to linear designs whereby electrodes are arranged along the vertical/axial dimension of implantation or to designs with small longitudinal spread, thus being unable to sample anatomical structures that span several millimeters along the horizontal axis. Indeed, mesoscale sampling of the LFP using 2D CMOS‐based probes with precise geometrical layout enabled insights into both intra‐ and translaminar features of the theta and gamma oscillatory dynamics in the hippocampus^[^ ^13^, ^14^, ^15^, ^16^ ^]^ and neocortex,^[^ ^17^ ^]^ decoding of the rat position from the spatio‐temporal features of theta oscillation waveshape,^[^ ^18^ ^]^ dissecting the role of different hippocampal fields to sharp wave/ripple generation^[^ ^19^ ^]^ and anatomical decomposition of the extrahippocampal inputs dynamics during sleep.^[^ ^20^, ^21^ ^]^ Combining continuous local and global dense recording would enable linking distributed large‐scale neural ensemble sampling to the multiregional oscillatory dynamics reflected by the detailed 2D sampling of the LFP.

The ability to densely sample extracellular signals for both single neuronal populations and local field potentials for assessing inter‐regional communication with appropriate anatomical resolution requires precise geometrical scaling of the CMOS‐based shanks. However, positioning and inserting multiple single‐shank electrode devices into the brain at a small pitch of a few hundred microns as required for local and global 2D sampling is a technically challenging task. Passive multi‐shank silicon probes with 96 or 256 recording electrodes can sample large tissue volumes with relatively low single‐unit yield due to low and 1D local electrode density.^[^ ^13^, ^22^ ^]^ Active 4‐shank probes (Neuropixels 2.0)^[^ ^12^ ^]^ can achieve local dense coverage, but these probes can only record from a subset of the available electrodes simultaneously, thus limiting the monitored area and providing uneven 2D continuous sampling at the circuit scale. Alternatively, multiple 4‐shank probes can be used to increase the number of recording channels. However, this introduces challenges for implantation in small rodents and does not allow for sampling neural activity at the full spatial resolution of the probe field‐of‐view.

To address this general problem related to any type of brain circuits in which vertical and horizontal information needs to be simultaneously sampled, here we present a novel CMOS‐probe that exploits the circuit modularity and the channels‐to‐electrode ratio of 1 of the SiNAPS technology.^[^ ^6^, ^23^ ^]^ This allows to replicate instances of 32 electrode‐pixel modules and achieves simultaneous recordings of 1024 electrode‐pixels distributed on eight shanks (pitch of 300 µm), covering an area of 2 by 2 mm (4.12 mm^2^) providing both dense local and large scale 2D sampling of the wide‐band extracellular signal. In vivo, recordings from the brains of behaving head‐fixed mice demonstrate how broadband neural signals can be acquired from the entire width of the hippocampus with a vertical coverage spanning from cortical to sub‐hippocampal regions. This novel CMOS probe allows the continuous monitoring at high spatial resolution (<30 µm center–center electrode pitch) of the 2D electro‐anatomy of the entire hippocampus with precise region and layer resolution. At the same time, the high density of the recording electrodes offers isolation of single neuron spikes and characterization of their spatio‐temporal profiles across the whole array. We demonstrate the capabilities of this 8‐shank 1024 channels active SiNAPS probe to sample multiple layers in the hippocampus and neocortex to link electro‐anatomically resolved oscillatory dynamics across the hippocampal circuits to distributed population activity of large populations of neurons.

## 2 Results

### 2.1 SiNAPS Multi‐Shank Electrode‐Pixel Probe for Large‐Area Intracortical Recordings

**Figure 1:**
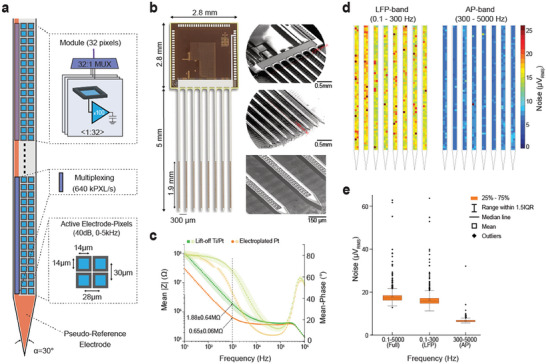
Layout and recording performances of the realized 8‐shank SiNAPS probe. a) Schematic view of the architecture and dimensions of a shank comprising four frontend modules of 32 active electrode‐pixels arranged over a 88 µm wide shank. Each module provides a 32:1 time division multiplexer operating at 640 000 electrode‐pixels per second (or 640kPX s^−1^). b) Optical view and Scanning Electron Micrographs (SEM) showing a structured 8‐shank SiNAPS probe at different magnification levels. c) Electrochemical Impedance Spectroscopy (EIS) of platinum electrodes in saline after the microstructuring post‐processing of the probes and after the electrochemical deposition of a Pt layer. The mean electrode impedance module at 1 kHz is 1.88 ± 0.64 MΩ/electrode and is reduced to a mean impedance of 0.65 ± 0.06 MΩ/electrode after electrodeposition (n = 10 probes × 1024 electrodes, mean ± SD). d) The noise of the electrode‐pixel circuits measured in saline for the full recording frequency band (Full‐band, 0.1–5000 Hz), the Local Field Potential frequency band (LFP‐band, 0.1–300 Hz), and the Action Potential frequency band (AP, 300–5000 Hz). The false color map on the top shows the homogeneity of the electrode‐pixels noise performances for the 1024 electrodes of the 8‐shank SiNAPS probe. e) Box‐plots quantify the root‐mean‐square noise values of 17.78 ± 3.4 µV_RMS_ (Full‐band), 16.45 ± 3.47 µV_RMS_ (LFP‐band) and 6.67 ± 1.02 µV_RMS_ (AP‐band) (n = 4 probes × 1024 electrodes, mean ± SD).

We realized an active dense electrode‐pixel probe with 8 shanks providing continuous intracortical electrical recordings from 1024 electrodes distributed over an area of 4.12 mm^2^ (**Figure** **1**). The CMOS circuit of this multi‐shank SiNAPS probe is based on a power‐efficient analog frontend electrode‐pixel module that was optimized from the one proposed in Angotzi et al. (2019).^[^ ^6^ ^]^ Each module comprises 32 electrode‐pixel sites with platinum (Pt) electrode square areas of 14 µm x 14 µm and microelectronic circuits beneath each electrode for in situ amplification and low‐pass filtering (Figure 1a; Figure S1, Supporting Information, Gain = 40 dB, f_−3_ _dB_ = 5 kHz). These 32 electrode‐pixels are time‐multiplexed in each module to an analog single output channel. An active feedback circuit corrects for potential DC drifts that arise at the tissue‐electrode interface and keeps the amplifier of each electrode‐pixel within its linear range of operation. The active feedback circuit is shared in a time‐division multiplexed fashion among the 32 electrode pixels of the module, and it periodically and automatically adjusts the working point of the in‐pixel low‐noise amplifiers. Such DC offset correction procedure was deemed favorable to integrate low‐area frontends in each electrode‐pixel, as it avoids the need for additional circuit elements, such as resistor‐capacitor (RC) circuits or large feedback capacitors that are typically required for AC‐coupled solutions. Feedback capacitors are subject to mismatch, can degrade the input impedance, and may lead to signal attenuation. Furthermore, recordings of ultraslow and DC signals may provide novel information about the physiological and pathological operations of brain circuits.

To minimize the probe width and consequent possible tissue damage, the analog frontend module arranges the 32 electrode pixels in a regular array with a center‐to‐center electrode pitch of ≤30 µm, and with a layout of two columns. This two‐column configuration yields a cross‐sectional shank width of 88 µm (Figure 1b) and a shank thickness of 50 µm. Each shank has a length of 5070 ± 10 µm and integrates four electrode‐pixel modules from the tip, for a total number of 128 electrode‐pixels and an active recording length of 1924 µm. The center‐to‐center separation among shanks is 300 µm, tailored for covering large portions of the mouse hippocampus with an 8‐shank probe defining an active recording area of 4.12 mm^2^. The electrode channel density computed by considering the overall area of this probe is close to 90 electrode‐channels mm^−2^, which is an order of magnitude denser than previously published CMOS probes. The electrode impedance was reduced to 650 ± 0.6 kΩ by electrodepositing a thin rough layer of platinum (Figure 1c). Together with circuit optimization with respect to noise, area and power consumption, we achieved a uniform low noise (Figure 1d,e) in the action potential (AP, 0.3–5 kHz) and local field potential (LFP, 0.1–300 Hz) frequency bands accounting for 6.67 ± 1.02 µV_RMS_ and 16.45 ± 3.47 µV_RMS_ root‐mean‐square (RMS), respectively, while consuming only 6 µW of power per electrode‐pixel. Each shank of the probe also integrates a Pt electrode covering the entire length of each shank and can be used as an on‐probe pseudo‐reference (or internal reference) expanding a total area for all shanks of ≈0.4 mm^2^.

### 2.2 In Vivo Validation of The Recording System and Estimation of the Signal Quality

To demonstrate the capabilities of the SiNAPS technology, we performed recordings in head‐fixed awake mice (n = 7 mice, Figures S2 and S3, Supporting Information). All probes were successfully used for recordings and showed homogeneous in‐vivo RMS signal values, without significant changes along and across recording sessions (Table S1, Supporting Information). The observed maximum number of non‐functional electrode‐pixel channels was less than 2%. As detailed in the Experimental Section, such non‐functionality is not necessarily related to a circuit or to an electrode failure, but rather due to the working principle of the autozeroing circuit of SiNAPS probes used to compensate DC offsets.^[^ ^6^ ^]^

**Figure 2:**
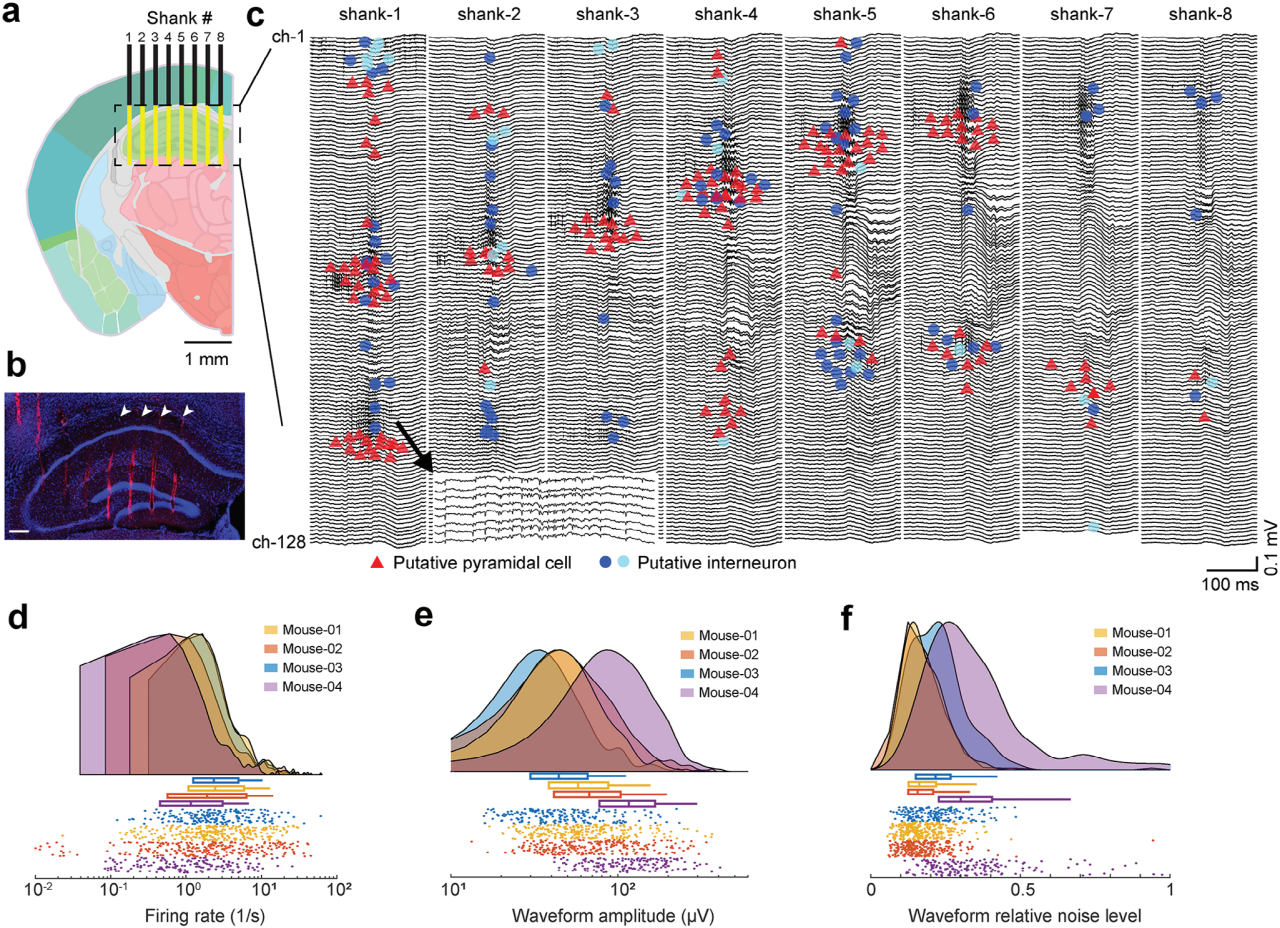
Multi‐regional recording in head‐fixed mice using SiNAPS electrode. a) The schematic of the ideal recording location in the hippocampus is overlaid on image 73 of the Allen Mouse Brain Atlas. The yellow area shows the location of recording electrodes spanning 2141 by 1924 µm (width, depth). b) Histological reconstruction of the probe tracks with 4′,6‐diamidino‐2‐phenylindole (DAPI; blue) and DiI (red) staining. White arrows show shanks 4–8. The scale bar is 250 µm. c) Wide‐band signal (0.1–5000 Hz) recorded on 1024 channels in a wild‐type mouse. The putative location of recorded neuron somata is overlaid on the LFP signal (n = 147 putative pyramidal cells, 94 narrow interneurons, and 19 wide interneurons represented by red, blue, and cyan, respectively). Neurons were clustered in the cellular layers of the cortex and hippocampus (ch‐1 represents the dorsal surface of the brain). Zoomed inset shows the spiking activity from shank‐1. d–f) Firing rate (d), waveform amplitude (e; trough‐to‐peak), and relative noise level (f; waveform standard deviation divided by the waveform amplitude) of hippocampal single units (n = 4 mice, one session per mouse).

**Figure 3:**
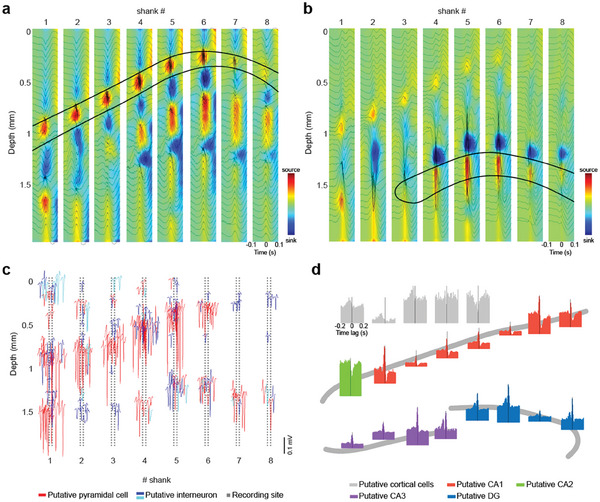
Electroanatomy of the hippocampus. a) Sharp wave ripple triggered LFP‐CSD map (n = 100 ripples, n = 64 channels per column of a shank). The average LFP signal of 32 channels (every second channel in one column) is overlaid on the CSD map. Note the characteristic negative SPW and sinks in the stratum radiatum accompanied by ripples and flanked by passive sources in the pyramidal layer and stratum lacunose‐molecular after the ripple peak. b) Dentate spike‐triggered LFP‐CSD map (n = 542 dentate spikes, n = 64 channels per column of a shank). The average LFP signal of 32 channels is overlaid on the CSD map. Note the characteristic source‐sink‐source configuration. Zero millimeters corresponds to the topmost channel on each shank. c) Simultaneously recorded well‐isolated single units recorded with SiNAPS electrode from the hippocampus of a head‐fixed, awake mouse. The mean waveform of each neuron is shown at the location of the maximum waveform amplitude (n = 260 putative single units, red is putative pyramidal cell, blue is putative narrow waveform and cyan is putative wide waveform interneuron). Zero corresponds to the location of the topmost channel of the shank. d) Separation of CA2 region based on peri‐ripple firing rate histograms of single units (ripples were detected in the pyramidal layer of CA1). Histograms are color‐coded to mark their regions of origin. Each histogram corresponds to a cluster of single units in c. Hippocampal histograms are remarkably similar with the exception of CA2 cells, allowing identification of this subregion.

The SiNAPS probe was inserted either in the hippocampus targeting CA1, CA3, and dentate gyrus subregions and the overlying cortex simultaneously (**Figure** **2**a,b) or in the frontal cortical areas targeting prelimbic, cingulate, and motor cortices (Figure S4, Supporting Information). Laminar LFP recordings allowed the identification of cell body and dendritic layers based on established electrophysiological markers (Figures 2c and **3**a,b). In addition to wide‐band LFPs, the low‐impedance electrodes provided high signal‐to‐noise recordings of extracellular spikes (82 ± 64 µV peak‐to‐peak voltage, mean ± SD; n = 5 mice, Figure 2d–f). The high density of electrodes enabled the recording and separation of spikes from individual neurons on multiple electrodes simultaneously (4–6 electrodes typically). This redundancy in the recorded signals can be exploited to improve the quality of spike sorting. After automatic clustering of recorded spikes using KiloSort2.5,^[^ ^24^ ^]^ we isolated 1300 neurons across 5 recording sessions (260 ± 94 single units/session, mean ± SD; n = 5 mice). These units were classified into putative cell types based on the waveform and spike train characteristics (Figure 2c, see Experimental Section for criteria of single unit classification).

To obtain valid LFP measurements, most neuroscience experiments use a reference screw above the cerebellum, considered to be the most electrically neutral part of the brain. We tested both platinum wire and stainless‐steel screw as reference electrodes and found that the signal quality did not depend on the material of the reference electrode. We also compared these external versus internal pseudo‐references in the same animal (Figure S5a, Supporting Information). Using the internal reference, each shank of the SiNAPS probe can act as a reference, creating a reference plane with a total area of 0.4 mm^2^. The single unit yield was similar regardless of the reference configuration (n = 57 and 58 single units using external and both references, respectively). We also tested whether the internal reference configuration could distort the LFP signal using visual evoked potentials. Recordings from primary and secondary visual cortices showed similar evoked responses and current sink and source distributions regardless of the reference choice (Figure S5b,c, Supporting Information).

### 2.3 Electroanatomy of Hippocampal Layers

The SiNAPS probe was designed to optimally record 2D laminar snapshots from brain structures such as multiple cortical or hippocampal layers. The recording channels span a brain area as large as 4.12 mm^2^ (2141 µm × 1924 µm). Simultaneous 1024 recording channels across 8 shanks allow the decomposition of the LFP signal into its individual sources. The relationship between afferents to dendrites and somata in the hippocampus and the characteristic depth profiles of various oscillatory LFP patterns can be used to identify hippocampal layers and their transitions.^[^ ^25^ ^]^ First, we detected sharp‐wave ripples (SWR) in the pyramidal layer of CA1^[^ ^26^ ^]^ and constructed a ripple‐triggered average current source density (CSD) map^[^ ^27^ ^]^ (Figure 3a). The 2D CSD map confirmed the source‐sink‐source distribution of SWRs corresponding to apical dendritic excitation of CA1 pyramidal neurons by the synchronous activity of CA3 neurons. To further differentiate the subregions of the hippocampus, we identified a channel in the hilus and in the molecular layer of the dentate gyrus manually and detected LFP dentate spikes (DS) automatically.^[^ ^28^ ^]^ We constructed a DS‐triggered average current source density (CSD) map which confirmed the source‐sink‐source configuration of the medial perforant pathway input from the medial entorhinal cortex innervating the dendrites of granular cells (Figure 3b). Utilizing the high yield of single units, we confirmed the soma location of the recorded neurons, estimated by the peak‐to‐peak waveform amplitude of each unit^[^ ^13^, ^29^ ^]^ (Figure 3c) and assigned to the recording electrode‐pixel of the relevant shank in the CSD map. The high number of single units also confirmed that large fractions of both pyramidal cells and interneurons fired synchronously during SWRs in the CA1 subregion but less so in the CA2 subregion^[^ ^19^ ^]^ (Figure 3d).

An alternative way to construct the electroanatomic map of the hippocampus is to exploit the observation that gamma oscillations are layer‐specific in the hippocampus and neocortex.^[^ ^16^, ^22^ ^]^ Within‐layer gamma coherence is always larger than across different layers due to the projection of the rhythmic inputs from upstream partners. Coherence maps in the broad gamma frequency band (30–90 Hz) were constructed between LFPs at manually selected electrode‐pixels in different layers (selected electrode‐pixels were chosen from the cortex, stratum oriens, pyramidale, radiatum of CA1 and stratum pyramidale of CA3, granule and molecular cell layer of DG) and the remaining 1023 channels. This procedure reliably outlined the anatomical boundaries in the cortex and subregions of the hippocampus (Figure S6, Supporting Information) and provided a 2D anatomical map of the hippocampus in vivo. The electroanatomic map constructed from combined unit firing and LFP signals corresponded faithfully to the histological reconstruction of the electrode tracks and the anatomic layers of the hippocampus (Figure 2).

**Figure 4:**
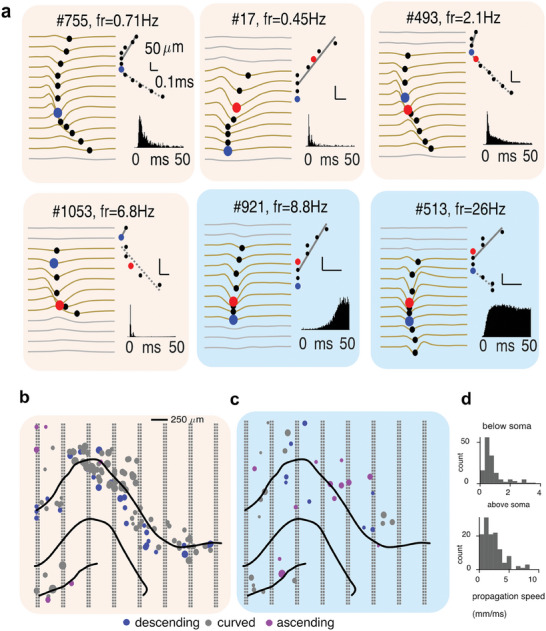
Topographic analysis of the action potential backpropagation. a) Examples of backpropagation of the action potentials (AP) in 4 putative pyramidal (orange shaded panels) and 2 putative inhibitory cells (blue shaded panels). Each panel shows the average AP waveform across channels surrounding the approximate soma location (left, black markers show spike troughs, red markers show troughs of the largest amplitude and blue markers show the earliest spike trough), quantification of the AP backpropagation speed and direction (right top) and the spiking autocorrelogram (right bottom). b) Depiction of the SiNAPS 8‐shank probe (gray dots) and the approximate anatomical location of the hippocampal layers (black curves) from an example recording session. Superimposed are approximated soma locations of all well‐isolated putative excitatory cells (left, orange shading, 118 cells) and inhibitory cells (right, blue shading, 38 cells) with different backpropagation characteristics (color: direction of backpropagation; marker size: speed). c) Population summary distributions for speed of AP backpropagation below (top) and above (bottom) the putative soma location.

To further exemplify the opportunities afforded by the dense and large area sampling of the neural tissue, we characterized patterns of action potential (AP) backpropagation across all hippocampal layers sampled by the 8‐shank SiNAPS probe (**Figure** **4**a). Such analysis can assist in further sub‐categorizations of neuronal types beyond putative excitatory versus inhibitory type^[^ ^30^ ^]^ by revealing local features of their main dendritic orientation, asymmetry of apical versus basal dendrites, and AP propagation speed with respect to their global circuit localization. Using the channel with the largest AP amplitude as the reference channel, a search was performed for above‐threshold troughs (>12% of the actual peak) on nearby electrodes (± 250 µm).^[^ ^31^ ^]^ Using the relative distance of the recording channels and the trough delays relative to the earliest trough, two linear fits were extracted for each cluster, one above and one below the putative soma location. The channel with the earliest peak was defined to be the putative AP generation site (typically the axon hillock). In many cases the earliest trough did not coincide with the largest amplitude AP, the latter being the conventionally assigned soma location. This may be explained by the axon hillock‐initiation area, producing an earlier depolarisation trough which is shortly followed by the full membrane depolarisation event. Using these linear fits, we classified backpropagation as ascending, descending, and or both (we termed as curved); a classification that reflects the underlying dendritic tree and its asymmetry. Interestingly, we found that not only putative pyramidal cells but also putative inhibitory neurons display diverse patterns of dendritic morphology, as reflected by the AP backpropagation (Figure 4b,c). The anatomical pattern for putative pyramidal cells shows that consistently across the transverse axis, descending AP backpropagating cells are mainly restricted to the superficial CA1 layer. At the same time, putative interneurons in dendritic domains showed highly variable direction of AP backpropagation. We found that AP backpropagation velocity was highly variable across cells and putative dendritic domains^[^ ^32^, ^33^ ^]^ (Figure 4d).

**Figure 5:**
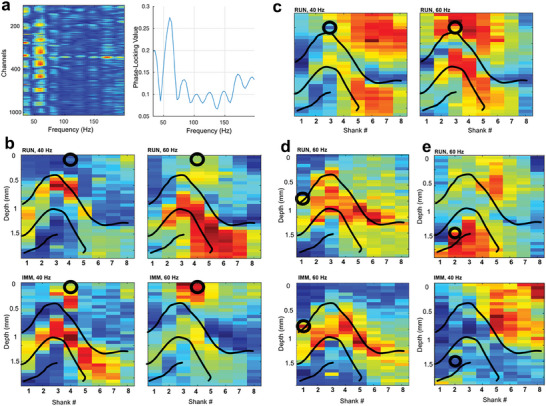
Topographic analysis of the phase‐locking of unit activity to the LFP. a) Example of the pseudo‐color‐coded strength of phase locking value (PLV) of a single interneuron (str. oriens) spiking to the LFP across all channels of the array (left) and to a best‐locked channel in CA1 str. rad. (right). b–e) Example color‐coded topographic PLC at various peak frequencies and different brain states for example neurons. Superimposed contours represent locations of the CA1 stratum pyramidale, DG granular layer, and CA3 stratum pyramidale. The black circle shows the approximate location of the unit cell body. Zero millimeters corresponds to the topmost channel on each shank. (b) PLV map of CA1 str. oriens putative interneuron at 40 Hz (left) and 60 Hz (right) during running (top, RUN) and immobility (bottom, IMM) states. (c) PLV map of CA1 putative pyramidal cell at 40 Hz (left) and 60 Hz (right) during running state. (d) PLV map for putative CA1 pyramidal cell at 60 Hz during running (top) and immobility (bottom) states. (e) PLV map for putative CA3 pyramidal cell at 60 Hz during running (top) state and 40 Hz during immobility (bottom) state.

Finally, we set out to utilize the two main design advantages of the SiNAPS arrays that stem from dense large‐scale 2D sampling, namely, the ability to isolate distributed anatomically localized neural ensembles and extract electro‐anatomical properties of oscillatory dynamics from 2D‐sampled laminar‐resolved LFP signal. To this end, we explored the laminar diversity of the hippocampal gamma oscillations through the prism of phase coupling of the spiking of hippocampal populations to the LFP signal across the array.^[^ ^13^, ^16^, ^17^, ^34^, ^35^ ^]^ Laminar‐localized oscillatory power in the gamma band of the LFP reflects synchronous synaptic inputs of the local or upstream gamma rhythm generators^[^ ^16^, ^17^, ^34^ ^]^ (Figure S5, Supporting Information), which results in phase‐coupling of the firing of neural ensembles both upstream, as part of the generator circuit, or downstream, as part of post‐synaptic population that is entrained by these gamma‐rhythmic inputs.^[^ ^35^ ^]^ Here we illustrate this approach and the capabilities of the SiNAPS probe to study circuit‐specific gamma oscillations via both pre‐ and post‐synaptic populations that they entrain. This was performed by computing the strength of phase‐locking of the firing of diversely located populations of single neurons to the LFP across the monitored hippocampal circuit during both locomotive and immobile states (**Figure** **5**). Single neurons showed a variable degree of phase‐locking (PLV) of their spiking activity to the LFP signal and gamma frequencies (Figure 5a), which reflect on the anatomical origin of the gamma oscillations observed in the LFP. Anatomical maps of the PLV at distinct peak PLV frequencies revealed laminar‐specific profiles that can be similar or different across frequencies and states (Figure 5b), showing similar or different putative gamma generators that these neurons are recruited by the respective frequency and state. We observed a variety of motifs with some neurons phase‐locking to distinct laminar patterns that are different between states at one frequency (Figure 5b, left) and similar at the other (Figure 5b, right). A single neuron could be coupled to the LFP in multiple anatomical domains and the magnitude of coupling could vary across these domains depending on the oscillation frequency (Figure 5c), be similar (Figure 5d), or combine common and different domains across frequencies and states (Figure 5e). Putative excitatory and inhibitory neurons localized in distinct hippocampal fields and reflected through 2D anatomical domains of their LFP phase‐coupling the “dictionary” of circuit‐specific gamma oscillations, that emerge transiently and recruit specific populations across all layers. Collectively obtaining these common anatomical motifs requires complex multivariate spectral analysis^[^ ^36^ ^]^ and will allow for extracting associated distributed populations that will enable uncovering the origin of the generator and the target populations it entrains.^[^ ^35^ ^]^

## 3 Discussion

As demonstrated in this work, SiNAPS probes with 1024 electrode‐pixels distributed across eight equally spaced shanks can record LFPs and APs concurrently from a large active recording area of ≈4 mm^2^. This extensive sampling of neural activity from large brain areas at the full spatial resolution of the probe (center‐to‐center electrode pitch ≤30 µm) yields a fine description of neural dynamics and population activity of single units across different brain regions and subregions while overcoming the drawbacks of passive neural probes such as large footprint, wiring and low‐density local sampling that is suboptimal for spike sorting.^[^ ^13^, ^22^, ^37^ ^]^ Here, we demonstrated SiNAPS multi‐shank probe recordings to study the close relationship between mesoscopic network dynamics, population activity of single neurons, and anatomy in the hippocampus. By continuously monitoring activity in the full 2D transverse extent of mice hippocampus, these high‐resolution electrical recordings can be used to generate extremely detailed 2D circuit‐level electro‐anatomical maps of the hippocampal neural dynamics and anatomically‐resolved characteristics of the spatio‐temporal backpropagation of individual neurons. To illustrate the advantage of joint dense local and global sampling by this SiNAPS probe, we characterized the topography of the gamma oscillatory dynamics via analysis of the phase locking of pyramidal and interneuron unit activity to LFPs for different frequencies and behavioral states.

Several different approaches have been recently proposed to target single neuron recordings from brain circuits with sub‐millisecond temporal resolutions. High channel count solutions based on the use of thin, flexible multi‐electrode polymer probes were recently proposed,^[^ ^38^, ^39^ ^]^ complementing the silicon‐based devices ecosystem. In addition to the clear advantages of the flexible substrate, silicon‐based technology enables higher density, and sampling resolution and allows for precise geometric relationships among all recording sites to attribute neural sources of neural dynamics to the boundaries of regions and sub‐regions in layered or deep structures such as neocortex or hippocampus and to examine functional connections within‐layer, across‐layers, and across‐regions with accurate co‐registration to their anatomy.^[^ ^14^, ^16^, ^17^, ^19^, ^22^, ^40^ ^]^

Active implantable probes based on CMOS technology surpass the limitations of passive probes, including vulnerability to electromagnetic interference, signal attenuation due to parasitic capacitances, and crosstalk between adjacent channels arising from densely packed metal lines. These include Neuropixels,^[^ ^4^ ^]^ Neuroseeker,^[^ ^7^ ^]^ and the SiNAPS active probes.^[^ ^6^ ^]^ While Neuropixel addresses the need to increase the number of read‐out channels by increasing the number of smaller front‐end circuits on the base of the probe,^[^ ^11^ ^]^ SiNAPS probes overcome the major scaling bottleneck caused by the spatial limits of analog frontends,^[^ ^41^ ^]^ thus allowing on‐shank multiplexing over modules of 32 in situ (i.e beneath each electrode) amplified and filtered electrode signals using a few, low impedance output lines robust to electromagnetic noise and cross‐talks. The measured frequency response of the in‐pixel amplifiers (Figure S1, Supporting Information), which also accounts for potential attenuation introduced by the electrode‐electrolyte interface, confirms their ability to capture both low‐frequency local field potentials (LFPs) and high‐frequency action potentials (spikes) with minimal signal distortion. The stable gain profile across the typical neuronal signal bandwidth ensures accurate signal transduction, allowing continuous full‐band monitoring of neural activity over the entire field of view and at the full spatial resolution of the probe. By increasing the number of recording sites while minimizing overall probe size, particularly the base area, SiNAPS assembled on smaller interfacing modules than that used in this work is also a promising solution for future chronic experiments in small laboratory animals, such as mice.

The key distinguishing features of the SiNAPS technology are: i) the use of the Active Pixel Sensor (APS) concept to realize an active dense CMOS probe with amplification and low‐pass filtering beneath each electrode site; ii) the modularity of the SiNAPS circuit architecture, based on modules of 32 electrode‐pixels that can be used to design probes with different layouts; iii) the integration of on‐probe time‐division multiplexing circuits within each module to continuously sample at 20kHz/electrode data from all electrodes on the probe; iv) the use of an autozeroing circuit (active feedback circuit for DC offset compensation) to implement a small‐area DC‐coupled frontend circuit, instead of an AC‐coupled frontend circuit; v) the high spatial resolution and channel density of the probe. Similar to Neuropixels and Neuroseeker devices, SiNAPS probes also use CMOS technology to integrate dense arrays of electrodes and read‐out circuits (Table S2, Supporting Information). However, SiNAPS grounds on a distinct circuit solution that uses the Active Pixel Sensor (APS) concept originally developed for image sensors,^[^ ^42^ ^]^ whereby active circuits for signal amplification, low‐pass filtering, and read‐out are located directly underneath each electrode‐pixel.^[^ ^43^ ^]^ This leads to distinct advantages. Notably, the SiNAPS approach allows integrating an equal number of front ends and electrodes as required for simultaneous recordings from the entire electrode array in contrast to other technologies where only a subset of channels can be simultaneously sampled. Consequently, the continuous and multi‐regional recording of both single neuronal activity and LFP dynamics is ensured at the level of each single trial and simplifies data analysis. It should be noted that by consuming only 6 µW of power per electrode‐pixel, SiNAPS probes do not cause noticeable tissue heating during operation.^[^ ^44^ ^]^ In addition, the unique modularity of the circuit architecture of SiNAPS probes allows the number of electrodes, shaft configurations from single to multiple shafts, and probe geometries to be scaled by replicating modules of 32 closely spaced electrode pixels, rapidly extending probe capabilities to meet specific experimental needs. Here, this feature was used to advance our 4‐shank layout anticipated in our pre‐print^[^ ^23^ ^]^ to an 8‐shank layout tailored to monitor the entire width of the hippocampus in behaving mice.

The multi‐shank SiNAPS technology advances the current tools that have been developed over the last few years for the post‐processing and interpretation of the rich spatio‐temporal content of data collected from high‐density neural probes.^[^ ^8^, ^9^, ^12^, ^45^, ^46^ ^]^ At the same SiNAPS probe recordings can be analyzed using computational tools established for other active dense probes.^[^ ^47^ ^]^ For instance, all existing solutions to the signal drift problem are not specific to a particular silicon probe technology and can exploit the small electrode pitch and a large number of electrode sites of SiNAPS probes to precisely track the population of the neurons that “drift” along the shank of the probe. Ultimately, this will lead to a better understanding of the circuit‐level mechanisms underlying distributed brain functions. In this work, we mainly used recordings from the hippocampus to illustrate the benefits of the new technology because of its well‐known laminar structure and a large body of background knowledge on both the population activity of single neurons and the large‐scale network dynamics, making it ideal to reveal the link between the two.

In this direction, here, we demonstrate the potential and utility of 2D recordings for precise layer identification, cell‐type characterization, and inter‐regional communication analysis. AP backpropagation features visualized across the hippocampal complex anatomy can provide insights into neuronal variability analogous to immunohistochemical or other cell labeling. While preliminary, our observations of distinct expression within CA1 sublayers of bidirectional versus apical‐only dendritic AP backpropagation might reflect important distinctions of the morphology and physiology of CA1 superficial and deep cells and their integration of respective entorhinal inputs.^[^ ^32^, ^48^ ^]^ It is likely that morphological differences map on functional ones, reflecting whether pyramidal cells are endowed with stronger integration and plasticity of basal or apical dendrites that receive CA2 and CA3 inputs, respectively. Further characteristics of the AP backpropagation, such as the behavior‐dependent variation of spatial extent and the velocity of the backpropagation might offer important information about in vivo dendritic electrogenesis.^[^ ^32^, ^33^ ^]^

A large number of anatomically resolved and well‐isolated putative pyramidal cells and interneurons offer inter‐regional analyses of input‐output circuit operations. One such promising method is relating spiking activity to the 2‐D LFP data,^[^ ^16^, ^17^ ^]^ potentially understanding the link between ensembles of single neurons and oscillatory dynamics within and across hippocampal regions in behaving animals. Laminar localization of hippocampal gamma oscillations in the LFP signal has been extensively documented and linked to the dynamics of local and upstream circuits.^[^ ^35^ ^]^ While anatomical profiling of the LFP oscillations is very powerful, it mainly focuses on large power laminar‐coherent and frequently occurring oscillations. At the same time spike‐LFP locking of specific neural populations, as shown here, can reveal localized and potentially transient oscillation generators. Future work is needed to expand spike‐LFP spectral analysis to multivariate cases, distinguishing rhythmic LFP patterns generated by presynaptic inputs causing locked cell firing^[^ ^25^ ^]^ or those caused by the output of the ensemble that a given cell belongs, as well as the contribution of local recurrent synaptic inputs and action potentials.^[^ ^49^ ^]^ Aggregation of single neurons into ensembles distributed across the hippocampal circuit and locked to oscillatory LFP in a specific anatomical domain can reveal oscillatory motifs with specific inputs, state correlates, and functional roles.^[^ ^50^ ^]^ We also show examples of cortical recordings and illustrate how a similar approach can be applied to discovering principles of interlayer and inter‐regional communication in the neocortex. The large span of the probe shanks also offers opportunities to study temporal interactions among distinct cortical columns in sensory areas, multiple subcortical regions, and nuclei where dense 2D electroanatomy allows online demarcation of the anatomical boundaries, such as in thalamic, basal ganglia and brain stem nuclei.^[^ ^51^, ^52^, ^53^ ^]^

Overall, SiNAPS probes can be used to explore the close relationship between distributed multiple single‐unit population activity, mesoscopic network dynamics, and functional connectivity. In perspective, upon miniaturization of packaging and development of flexible tether SiNAPS electrodes can be readily used in chronic applications.

## 4 Experimental Section

### Fabrication of SiNAPS Probes

The multi‐shanks SiNAPS probes were realized in a standard 180 nm Complementary Metal‐Oxide Semiconductor (CMOS) technology. The microstructure process was first established for 4‐shank probe prototypes at the dye level and then scaled to wafer‐level post‐processing with similar processes (Corticale Srl, Italy) for 8‐shank probes. Prototyping devices were delivered from the CMOS foundry as single dies with the size of (W × L × T = 3 mm × 8.5 mm × 152.4 µm) and were post‐processed in the IIT cleanroom facility to modify the electrode material and to structure thin probes. The first post‐processing step was used to structure a noble metal (Pt) layer on top of the native CMOS metal of each electrode‐pixel. The top CMOS insulator was opened at a size of 10 × 10 µm2, and the Pt layer was structured with a size of 14 × 14 µm2 to guarantee complete coverage. The second post‐processing step used reactive ion‐etching of silicon to structure the probe shape and a backside grinding process to thin the probes to a final thickness of 50 µm. To reduce the electrode impedance, a rough layer of Pt was electroplated on the Pt electrodes using a potentiostat/galvanostat (PG‐STAT204, Metrohm Autolab, Switzerland) and a three electrodes electrochemical cell (Ag/AgCl reference electrode, Pt counter electrode, and the short‐circuited 1024 electrodes of the SiNAPS probe as working electrode). Electrochemical stripping in sulfuric acid (0.5 mol l^−1^) was used to clean the electrodes. Then, Pt was electroplated using a commercial solution (“Platinum AP + 4G/L Pt”, Technic, Italy) and by applying a current of 10 nA/electrode for 1 h. After post‐processing, SiNAPS probes were mounted on a printed circuit board (PCB) and wire bonded for interfacing with the SiNAPS recording system (Figure S8, Supporting Information). SiNAPS probes were wire bonded using Au wires of 18 µm in diameter and protected with a thermally curable resin (Ostemer 322 Crystal Clear, from Mercene Labs). Other components on the PCB were the probe connector and components for electrical decoupling.

### Electrical and Electrochemical Characterization

Prior to in vivo recordings, the realized SiNAPS probes were tested with respect to their electrical and electrochemical performances. For the electrical characterization, the noise contribution of the electronic circuits was measured in dry conditions by setting the switch that was integrated into each electrode‐pixels to connect all the electrodes to a common ground. The noise of the electrode‐pixels was then measured in wet conditions by dipping the SiNAPS probes in a beaker containing a saline solution (NaCl 0.9%). For both conditions, data collected at the output of the whole signal acquisition chain of the probe was used to compute the power spectral density from which the root mean square noise contribution was quantified within specific frequency bands. To assess the capability of the in‐pixel amplifiers to acquire broad‐band electrical activity, their frequency response was also measured in wet conditions by using a platinum wire to inject pure sine waves of varying frequencies, ranging from 0.1 Hz to 10 kHz. Data collected in this way, which also included contributions from the electrode‐electrolyte interface, was used to measure the mean gain between 0.3 Hz and 3 kHz (Figure S1, Supporting Information).

Electrochemical impedance spectroscopy (EIS) was used to quantify the electrode impedance in saline solution using a potentiostat/galvanostat (PG‐STAT204, Metrohm Autolab, Switzerland). Since electrodes cannot be individually connected for the measure, the impedance value for the single electrode was estimated as 1024× larger than the one measured from all electrodes using an three electrodes electrochemical cell (Ag/AgCl reference electrode, Pt counter electrode, and the short‐circuited 1024 electrodes of the SiNAPS probe as working electrode). The module of the impedance for a single electrode at 1 kHz was then computed by dividing the measured value by the number of electrodes.

### SiNAPS Data Acquisition System

Electrophysiological data were collected from SiNAPS probes using two different instruments for experiments performed at LMU and NYU, respectively. In the first case, the SiNAPS Research Platform (IIT, Italy), while in the second case, a commercially available data acquisition system (SmartBox Pro, Neuronexus, USA) was used. Both instruments allowed to control SiNAPS probes and to sample neural data at 20 kSample s^−1^, with a 12‐bit resolution over a 2.4 V dynamic range. The use of two different systems in different laboratories strengthened the reproducibility of the SiNAPS probes' recording performances.

### SiNAPS Data Acquisition System—Experiments at LMU

These experiments were performed using the SiNAPS Research Platform (Figure S7, Supporting Information), which included a data acquisition board, a digital control unit, a custom coax cable assembly with embedded electronics, and data acquisition software running on a PC equipped with a Camera Link frame grabber (Grablink Base, Euresys, Belgium). The data acquisition board provided a bank of 32 analog to digital converters (MAX11105, Maxim Integrated, USA), one for each of the 32 electrode‐pixels modules of the SiNAPS probe. The digital control unit was built on an Opal Kelly ZEM4310 integration module based on an Altera Cyclone IV FPGA. The firmware of the digital control unit ensured the correct operation of the SiNAPS probe and data communication. This included the SiNAPS IP core (Corticale, Italy) that provided control signals, a bank of programmable bandpass filters (second order FIR filters with programmable high pass cut‐off of 2 Hz or 300 Hz and fixed low pass cutoff frequency of 5 kHz) and an automated calibration procedure to set the optimal voltage biasing^[^ ^54^ ^]^ of the SiNAPS probes prior to recordings. The custom coax cable assembly (1m long) connected the data acquisition board with the head‐stage mounting the SiNAPS probe. This assembly also integrated components for supplying the 1.8 V nominal power to the probe and included a multichannel digital to analog converter (12 bit over 1.8 V range) to generate the analog voltage biases set by the calibration procedure. The PC ran data acquisition software (SiNAPS Control Software, IIT, Italy) for data visualization and storage. This was developed in Microsoft Visual Studio Enterprise 2019 (Ver. 16.11.36, Microsoft Corp, USA) using the general‐purpose high‐level C# programming language and the. NET Framework 4.8.1 (Microsoft Corp, USA). Differently from the SmartBox Pro, the SiNAPS Research Platform concurrently stores two datastreams, namely electrode‐pixel data processed in real‐time by the FPGA, as well as a datastream of unprocessed raw data. This raw data was used for offline signal processing to obtain electrode signals with a frequency bandwidth ranging from 0.1 Hz to 5 kHz.

### SiNAPS Data Acquisition System—Experiments at NYU

Experiments were performed using the commercial system available from Neuronexus implements similar functionalities through the SmartBox Pro and the SiNAPS Interface Box (https://www.neuronexus.com/products/sinaps‐interface‐box/) which were interconnected through a standard HDMI cable. As for the SiNAPS Research Platform, data from each available electrode‐pixel was acquired and digitized at 20 kS s^−1^, with a 12‐bit resolution over a 2.4 dynamic range through the SiNAPS Interface Box which also implemented the same SiNAPS IP core (Corticale, Italy) and outputs electrode‐pixel data processed in real‐time by the FPGA (i.e., signals filtered over 2 Hz or 300 Hz to 5 kHz). A single HDMI cable was used to transmit in real‐time the digitized and filtered data from the SiNAPS BOX Interface to the SmartBox Pro which, in turn, implemented a high‐rate data transmission to the PC, where data was stored, analyzed, and visualized by the Allego Software (Neuronexus, USA).

For both systems, it has to be noted that for proper operation of the electrode‐pixels frontend circuit, SiNAPS probes needed calibration for setting an internal voltage bias at the beginning of a recording session. This automated procedure was implemented in the FPGA by the SiNAPS IP core (Corticale, Italy) and set most of the electrode‐pixel frontend circuits to operate the autozeroing circuit within the DC compensation range (≈160 mV) of the circuit. However, some electrode‐pixels might fall too close to the compensation limits and will be non‐functional for the specific recording session.

### Animal Experiments—Experiments at NYU

All experiments were performed in accordance with the Institutional Animal Care and Use Committee at New York University Medical Center (license # IA15‐01466). All efforts were made to minimize the number of animals used and the incurred discomfort. Animals were handled daily and accommodated to the experimenter before the surgery and head‐fixed recording. Mice (adult male n = 5 C57/Bl6 mice, 26–31 g) were kept in a vivarium on a 12 h light/dark cycle and were housed two per cage before surgery and individually after it. Atropine (0.05 mg kg^−1^, s.c.) was administered after isoflurane anesthesia induction to reduce saliva production. The body temperature was monitored and kept constant at 36–37 °C with a DC temperature controller. Stages of anesthesia were maintained by confirming the lack of a nociceptive reflex. The skin of the head was shaved, and the surface of the skull was cleaned by hydrogen peroxide (2%). A custom 3D‐printed headpost^[^ ^55^ ^]^ (Form2 printer, FormLabs, Sommerville, MA) or aluminum headpost and 3D‐printed recording chamber (LMU) was attached to the skull using C&B Metabond dental cement (Parkell, Edgewood, NY). The location of the craniotomy was marked and a stainless‐steel ground screw with a header pin was placed above the cerebellum. Each animal recovered for at least 7 days prior to habituation of the head‐fixation. Animals were allowed to walk freely either on a treadmill (NYU, Figure S2a, Supporting Information, https://github.com/misiVoroslakos/3D_printed_designs/tree/main/Treadmill_Rinberg) during recording sessions. The day before recording, a craniotomy was performed (2 mm posterior from Bregma and 1.5 mm lateral to midline targeting hippocampus and 2 mm anterior from Bregma and 0.5 mm lateral to midline targeting midline cortices) and the dura was removed. After the surgery, the craniotomy was sealed with Kwik–Sil (World Precision Instruments, Sarasota, FL) until the recording. On the day of the recording the animal was head‐fixed, the craniotomy was cleaned and the headpost was filled with sterile saline. The ground of the probe PCB was connected to the header pin and the probe was inserted into the target depth using a manual micromanipulator (MM‐33, Sutter Instruments, Novato, CA). The electrophysiological signal and transistor bias were constantly monitored during insertion. The collected data was digitized at 20 kS s^−1^ using Smartbox Pro and Activus SiNAPS interface box and visualized using Radiens Allego software (NeuroNexus Technologies, Ann Arbor, MI) (NYU). At least 30 min were waited after reaching the target depth before starting the data acquisition. 2 h sessions were recorded from each mouse. After the recording session, the craniotomy was sealed with Kwik–Sil and the animal was put back into its homecage. If more than one session was recorded from an animal, a new craniotomy was prepared as described above on the contralateral side.

### Animal Experiments—Experiments at LMU

All experiments were performed in accordance with the European Communities Directive 2010/63/EC and the German Law for Protection of Animals (TierSchG) and were approved by local authorities (license # ROB‐55.2‐2532.Vet_02‐22‐22). Animals (C57/Bl6 mice, n = 2, 3–5 months of age) had been trained to navigate a virtual linear track to receive a 5 µl sucrose/water (1% w/v) reward after each trial completion (Figure S3, Supporting Information). Once stable performance was achieved, the animals underwent stereotaxic surgery. Following induction of deep anesthesia with intraperitoneal injection of MMF (0.05mg kg^−1^ Medetomidine, 5mg kg^−1^ Midazolam, and 0.5 mg kg^−1^ Fentanyl) and maintenance of anesthesia using isoflurane (0.5% v/v in oxygen) the animals were equipped with a custom, lightweight, 3D‐printed recording chamber above the exposed skull (sealed with a thin layer of dental acrylic) as well as an aluminum head‐post. Stainless steel and Ag/AgCl ground/reference electrodes were placed above the cerebellum and PFC. Additionally, a 2.2 mm long and 3.5 mm wide cranial window above the left hippocampus (AP ‐2.3 mm) was established, the dura was removed and the window was sealed with Kwik‐Cast (World Precision Instruments Germany GmbH). During the surgery, the body temperature was monitored and kept constant at 37.5 °C using a homeothermic blanket. The animals were allowed to recover for at least 7 days (3 days of post‐operative care, Enrofloxacin, and Metacam) with food and water ad libitum. On the day of the recording, the animals were placed on the running wheel and their head was kept firmly via the head‐post and an additional adjustable articulated mount attached to the front of the recording chamber. The Kwik‐Cast seal was carefully removed, and the implantation site was inspected for possible re‐growth. The exposed brain surface was kept moist with warm saline. The SiNAPS probe was lowered using a linear nanopositioner (PI Q‐531.330 and PI E‐873 Q‐Motion Servo Controller) under tight visual control at a speed of 2–5 µm s^−1^. The probe's position was allowed to settle for at least 20 min before bias voltage adjustments could begin. The bias voltage was set to the highest possible value where no channel was saturated and subsequently decreased again by 5–10 mV. If after 15 min of waiting time, the signal quality required additional gain adjustments, this step was repeated, otherwise the recording was started. The collected data was digitized at 20 kS s^−1^ using custom acquisition hardware and software.

Both at LMU and NYU, SiNAPS probes were reused for different recording sessions (up to 5 times). To extend the lifetime of SiNAPS probes a cleaning procedure was adopted. In short, the probes were cleaned after each experiment using 1% Tergazyme solution for 3 h and distilled water for 10 h (the probe was soaked in these liquids). Tergazyme was a concentrated, anionic detergent with protease enzyme that was routinely used to clean rigid silicon probes, including Neuropixels probes.

Concerning the reference electrode, in two animals it was observed that when the reference electrode was not set correctly during the implantation surgery, this led to a high number of non‐functional electrode pixels (e.g., hundreds). Inserting a new reference electrode over the cerebellum resolved this issue.

### Visual Evoked Potential in Head‐Fixed Mice

The pupil was dilated using 1% atropine sulfate eye drops. The room lights were turned off to allow dark adaptation for 20 min. 5 ms light pulses were delivered using an LED light source (#Fortoo‐12V‐PMMA008, Fortoo), and 200 light pulses were delivered at 0.2 Hz using an Arduino UNO. The visual stimuli were delivered while the electrophysiology signal was collected using internal reference and then external reference configuration. The probe was kept at the same dorsoventral location for both measurements.

### Single Unit Analysis

A concatenated signal file was prepared by merging all recordings from a single animal from a single day. To improve the efficacy of spike sorting, stimulation‐induced onset and offset artifacts were removed before automatic spike sorting (1 ms before and 5 ms after the detected artifacts, linear interpolation between timestamps). Putative single units were first sorted using Kilosort 2.5^[^ ^24^ ^]^ which used drift‐correction to change templates continuously as a function of drift, and then manually curated the data using Phy2 (https://phy‐contrib.readthedocs.io/).

### Cell Type Classification

In the processing pipeline, cells were classified into three putative cell types: narrow interneurons, wide interneurons, and pyramidal cells. Interneurons were selected by 2 separate criteria; narrow interneurons were assigned if the waveform trough‐to‐peak latency was less than 0.425 ms. Wide interneuron was assigned if the waveform trough‐to‐peak latency was more than 0.425 ms and the rise time of the autocorrelation histogram was more than 6 ms. The remaining cells were assigned as pyramidal cells. Autocorrelation histograms were fitted with a triple exponential equation to supplement the classical, waveform feature‐based single unit classification (https://cellexplorer.org/pipeline/cell‐type‐classification/).^[^ ^56^ ^]^ Bursts were defined as groups of spikes with interspike intervals < 9 ms.

### Local Field Potential Analysis

Ripple detection and wavelet spectrogram calculation were performed as previously described.^[^ ^26^ ^]^ To detect ripples a single electrode in the middle of the pyramidal layer was selected. The wide‐band LFP signal was band‐pass filtered (difference‐of‐Gaussians; zero‐lag, linear phase FIR), and instantaneous power was computed by clipping at 4 SD, rectified and low‐pass filtered. The low‐pass filter cut‐off was at a frequency corresponding to p cycles of the mean band‐pass (for 80–250 Hz band‐pass, the low‐pass was 55 Hz). Subsequently, the power of the non‐clipped signal was computed, and all events exceeding 4 SD from the mean were detected. Events were then expanded until the (non‐clipped) power fell below 1 SD; short events (< 15 ms) were discarded. The pyramidal layer of the CA1 region was identified physiologically by increased unit activity and characteristic LFP patterns. The identification of dendritic sublayers was achieved by the application of current source density and independent component analysis to the LFPs.^[^ ^16^, ^40^, ^57^, ^58^ ^]^ To detect dentate spikes, the LFP recorded from a hilus and a molecular layer location was bandpass filtered (2–50 Hz). A positive deflection of the LFP difference between the channels in the hilus and molecular layer was detected. If the mean LFP value at the molecular layer during the dentate spike was lower than the baseline value (−36 to −16 ms) by > 0.19 mV, the dentate spike passed the criteria and was included for later analysis. The peak time of the dentate spike was defined as the time when the dentate hilus wide‐band LFP showed maximum value.^[^ ^30^ ^]^

### Backpropagation Analysis

The regularly spaced and dense electrode arrays allowed quantification of backpropagation properties of action potentials. Following clustering using Kilosort 2.5 and manual curation, backpropagation was assessed by first performing a search for above‐threshold waveforms on electrodes near the cluster's dominant channel, i.e., the channel with the largest spike amplitude approximating the soma location. The threshold was set to 12% of the minimum trough amplitude.^[^ ^31^ ^]^ In some cases, troughs were detected at time points earlier than that of the channel with the largest spike amplitude. In such cases, the channel with the earliest peak was deemed to initiate the action potential. Finally, the selected channels’ locations and corresponding trough delays were fitted with linear regression to extract the slopes, which corresponded to the inverse of the speed of backpropagation in time per unit distance (ms mm^−1^). In cases where distinct slopes were detected above and below the putative soma location, two slopes were extracted accordingly. Cells were classified into those with backpropagation being above (ascending), below (descending), or both (curved).

### Mapping of the Spike‐LFP Gamma Oscillation Coupling

Coupling of the spiking of single neurons to the LFP on each channel and at diverse gamma frequencies (30–200 Hz) was estimated using PLV statistics, i.e., the resultant length of the ensemble of phase values derived from the instantaneous phase of the band‐pass filtered LFP signal in a 5 Hz band centered on the respective frequency of interest. For smoothing and visualization purposes the adjacent four pixels within each location were averaged. This analysis was performed on raw data that were not processed by the real‐time hardware, but offline. Raw electrode signals acquired with the SiNAPS Research Platform were post‐processed to remove signal discontinuities associated with the regular calibration of the in‐pixel DC frontend and to filter electrode signals in a frequency bandwidth ranging from 0.1 Hz to 5 kHz.

### Coherence Maps in the Gamma Frequency

The LFP signals were filtered by a Gaussian band‐pass filter (from 30 to 90 Hz), and coherence maps were constructed between manually selected LFPs at reference sites in different layers and the remaining 1023 channels (n = 100 1 s intervals during sharp wave ripples). Reference sites were: 2‐2 channels in the cortex and stratum pyramidale of CA3, one channel in stratum oriens, pyramidale and radiatum of CA1, granule cell and molecular layer of dentate gyrus). This procedure reliably outlined the anatomical layer boundaries across layers of the hippocampus and across regions (cortex and hippocampus).^[^ ^16^ ^]^ As for mapping spike‐LFP gamma oscillation coupling, this analysis was performed on offline processed raw electrode signals that were acquired with the SiNAPS Research Platform.

### Statistical Analysis

Statistical analyses were performed with MATLAB functions or custom‐made scripts. No specific analysis was used to estimate a minimal population sample or group size, but the number of animals, sessions, and recorded cells were larger or similar to those employed in previous related works.^[^ ^16^, ^22^, ^40^ ^]^ The unit of analysis was typically identified as single neurons. In a few cases, the unit of analysis was sessions or animals, and this was stated in the text. Unless otherwise noted, a non‐parametric two‐tailed Wilcoxon rank‐sum (equivalent to Mann–Whitney *u*‐test) or Wilcoxon signed‐rank test was used. For multiple comparisons following ANOVA, Tukey's honesty post‐hoc test was employed. On box plots, the central mark indicated the median, bottom and top edges of the box indicate the 25th and 75th percentiles, respectively, and whiskers extended to the most extreme data points not considered outliers. Outliers were not displayed in some plots but were included in statistical analysis.

## Conflict of Interest

L.B, G.N.A and F.B have personal financial interests as they are shareholders of Corticale Srl. G.N.A and F.B. have employment financial interests in Corticale Srl, and are CSO and CTO respectively. All other co‐authors declare that they have no competing interests.

## Author Contributions

G.N.A., M.V., and N.P. contributed equally to this work. L.B., M.V., A.S., and G.B., wrote the manuscript. L.B. and G.N.A. coordinated the development of the SiNAPS probe technology. A.S. and L.B. designed the concept for the technology specifications. M.V. and A.S. designed the experimental study. G.N.A., F.B., and J.F.R. developed the multi‐shank SiNAPS probes and G.N.A. back‐end acquisition system. J.F.R., A.L., and L.B. developed the CMOS post‐processing of the first prototypes and J.F.R. and G.O. performed the electrochemical Pt deposition and characterization of probes realized by Corticale Srl. A.G., N.P., M.V., D.A., and G.S. designed and performed the animal experiments. With M.V. they analyzed data and contributed to the manuscript. F.B., D.A., G.N.A., and J.F.R. contributed to the animal experiments and to data analysis. All co‐authors reviewed the paper.

## Supporting information



Supporting Information

## Data Availability

The dataset will be available from the Buzsaki‐lab data bank55, (https://buzsakilab.com/wp/database/) and G‐Node repository (https://gin.g‐node.org/antsiro/SiNAPS_8shank). MATLAB script packages used in the analysis of this study can be downloaded from https://github.com/MouseLand/Kilosort, https://phy.readthedocs.io/en/latest/ and https://github.com/buzsakilab/buzcode.
