# Supplementary material for: Multi‐Shank 1024 Channels Active SiNAPS Probe for Large Multi‐Regional Topographical Electrophysiological Mapping of Neural Dynamics
Source: Adv Sci (Weinh). 2025 Feb 27;12(16):2416239. doi: 10.1002/advs.202416239 (PMC12021112; doi:10.1002/advs.202416239)
Supplement: Supplementary file 1 — Supporting Information [file ADVS-12-2416239-s001.docx]

**Supplementary Material for**

**Multi-shank 1024 channels active SiNAPS probe for large multi-regional topographical electrophysiological mapping of neural dynamics**

*Gian Nicola Angotzi^1,2,†^, Mihály Vöröslakos^4,†^, Nikolas Perentos^6,3,†^, Joao Filipe Ribeiro^1^, Matteo Vincenzi^1^, Fabio Boi^1,2^, Aziliz Lecomte^7^, Gabor Orban^1^, Andreas Genewsky^3^, Gerrit Schwesig^3^, Deren Aykan^4^, György Buzsáki^4,5^, Anton Sirota^3,*^, Luca Berdondini^1,*^*

^1^ Fondazione Istituto Italiano di Tecnologia, Microtechnology for Neuroelectronics Unit (NetS^3^ lab), Genova, Italy.

^2^ Corticale Srl, Genova, Italy.

^3^ Ludwig-Maximilians-Universitat, Faculty of Biology, Munchen, Germany.

^4^ Neuroscience Institute, Grossman School of Medicine, New York University, New York, NY, USA.

^5^ Department of Neurology, Grossman School of Medicine, New York University, New York, NY, USA.

^6^ University of Nicosia School of Veterinary Medicine, 2414, Nicosia, Cyprus

^†^ These three authors contributed equally to this work.

^*^ Co-last and co-corresponding authors.

Correspondance: sirota@bio.lmu.de, Luca.Berdondini@iit.it





**Figure S1**. **Measured gain-frequency response of the integrated electrode-pixel circuit**. The gain for the 1024 electrodes was characterized by immersing the 8-shank SiNAPS probe into a beaker containing phosphate-buffered saline (PBS). Pure sine waves of varying frequencies, ranging from 0.1 Hz to 10 kHz, were injected using a platinum wire. The mean gain between 0.3Hz and 3kHz is of 40.35dB with sigma 0.41.


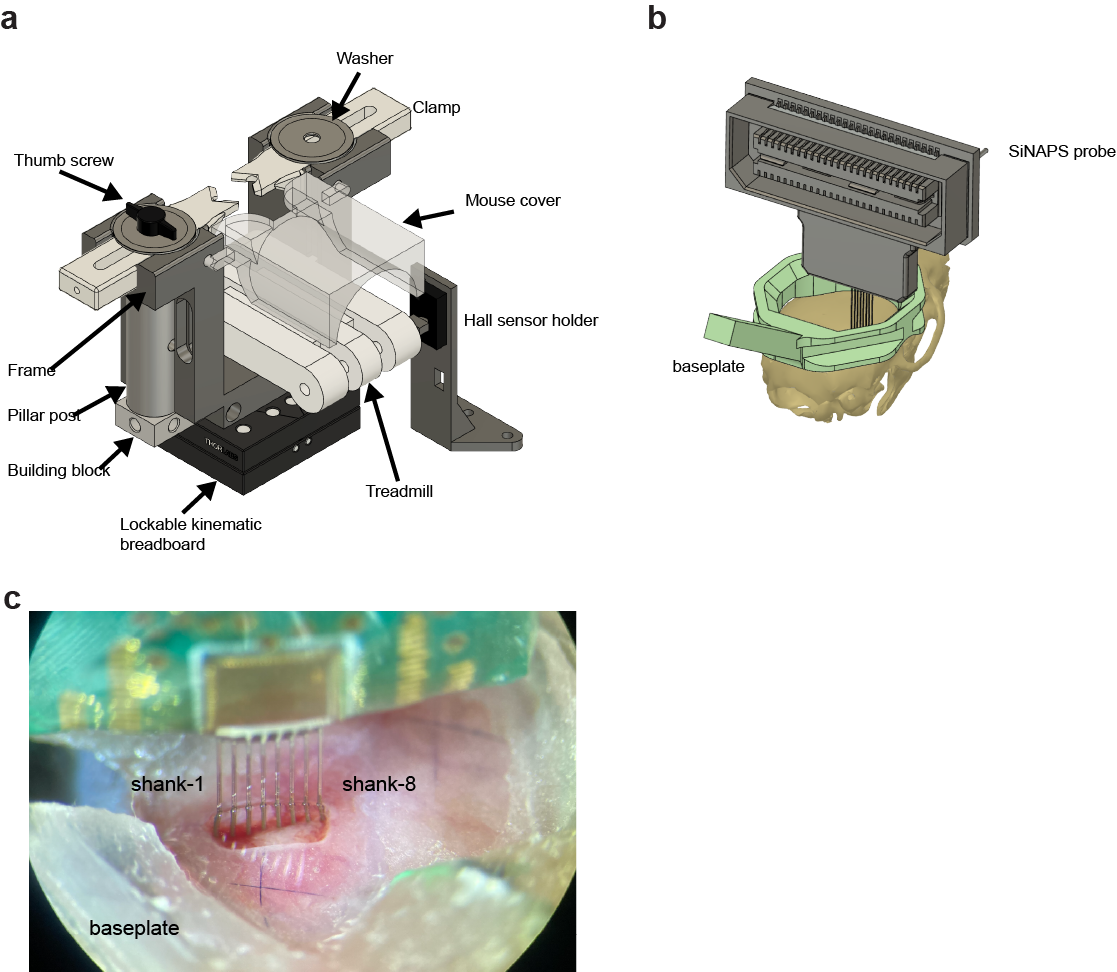


**Figure S2. Experimental setup for head-fixed, awake experiments (NYU). (a)** CAD design of the assembled treadmill system. Mice were head-fixed and were allowed to freely move on the treadmill during recording sessions. **(b)** 3D-printed base plate was attached to the skull to fix the animal (green part). PCB of the SiNAPS electrode is shown in scale relative to the mouse’s skull. **(c)** Photograph of the insertion of SiNAPS electrode into the left hippocampus.


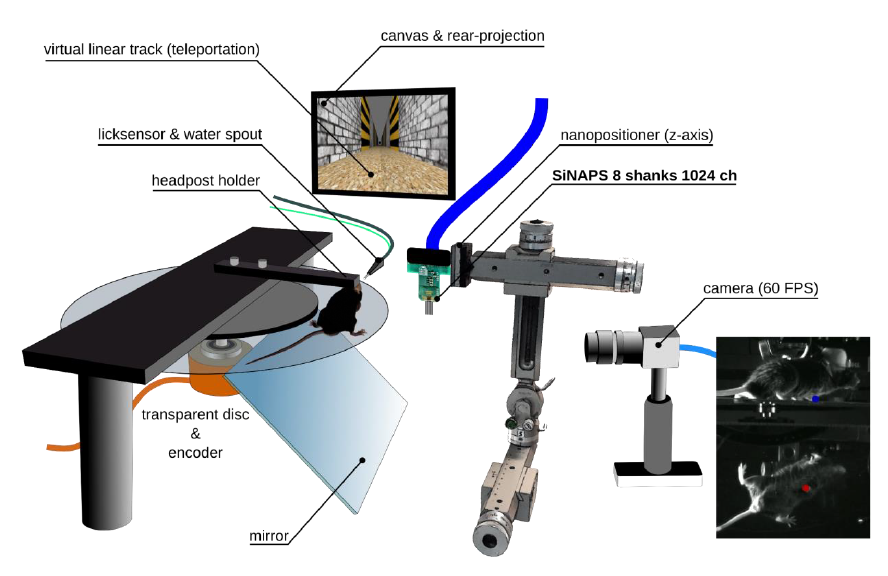


**Figure S3. Experimental setup for head-fixed, awake experiments (LMU)** Water-deprived mice were trained to navigate through a virtual linear track for water reward.

**
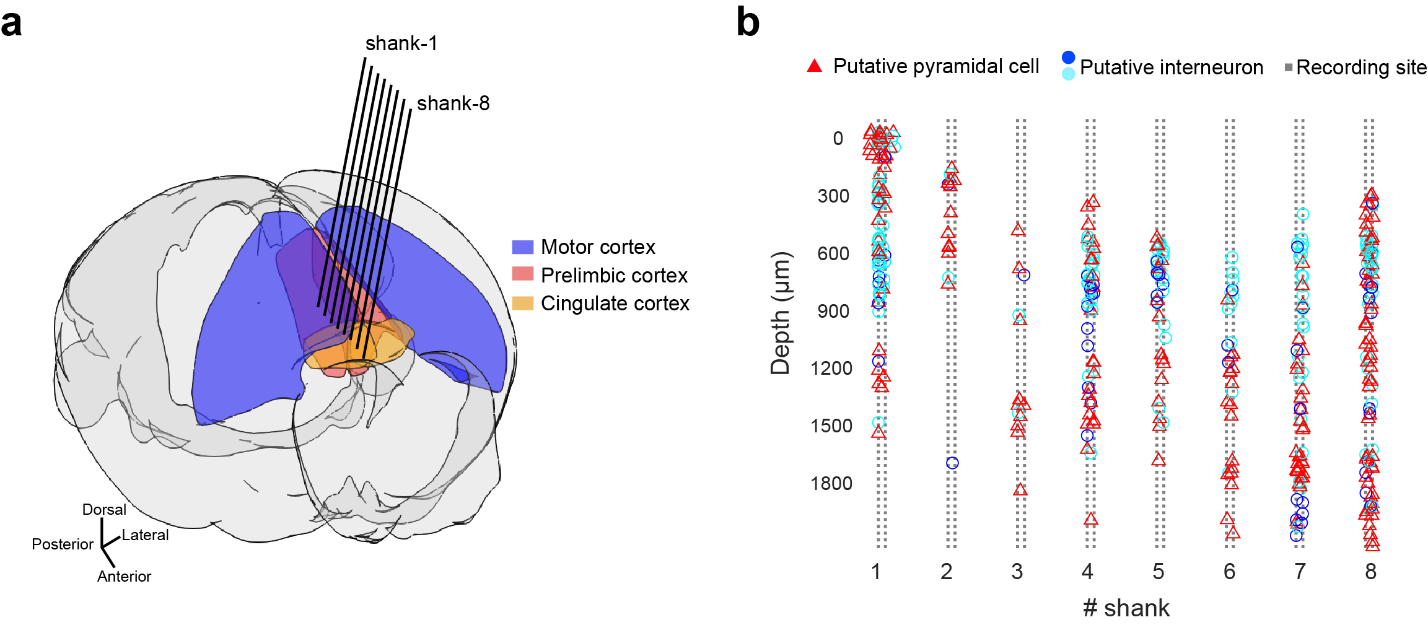
**

**Figure S4. Recordings from prefrontal cortices using SiNAPS electrode. (a)** Schematic location of SiNAPS electrode recording from motor (blue), prelimbic (red) and cingulate cortices (yellow)^1^. **(b)** Probe layout (grey rectangle) is shown with the putative location of recorded neuron somata (n = 408 neurons, n = 215 putative pyramidal cells, 55 narrow interneurons and 138 wide interneurons). 0 μm represents the brain surface.

**
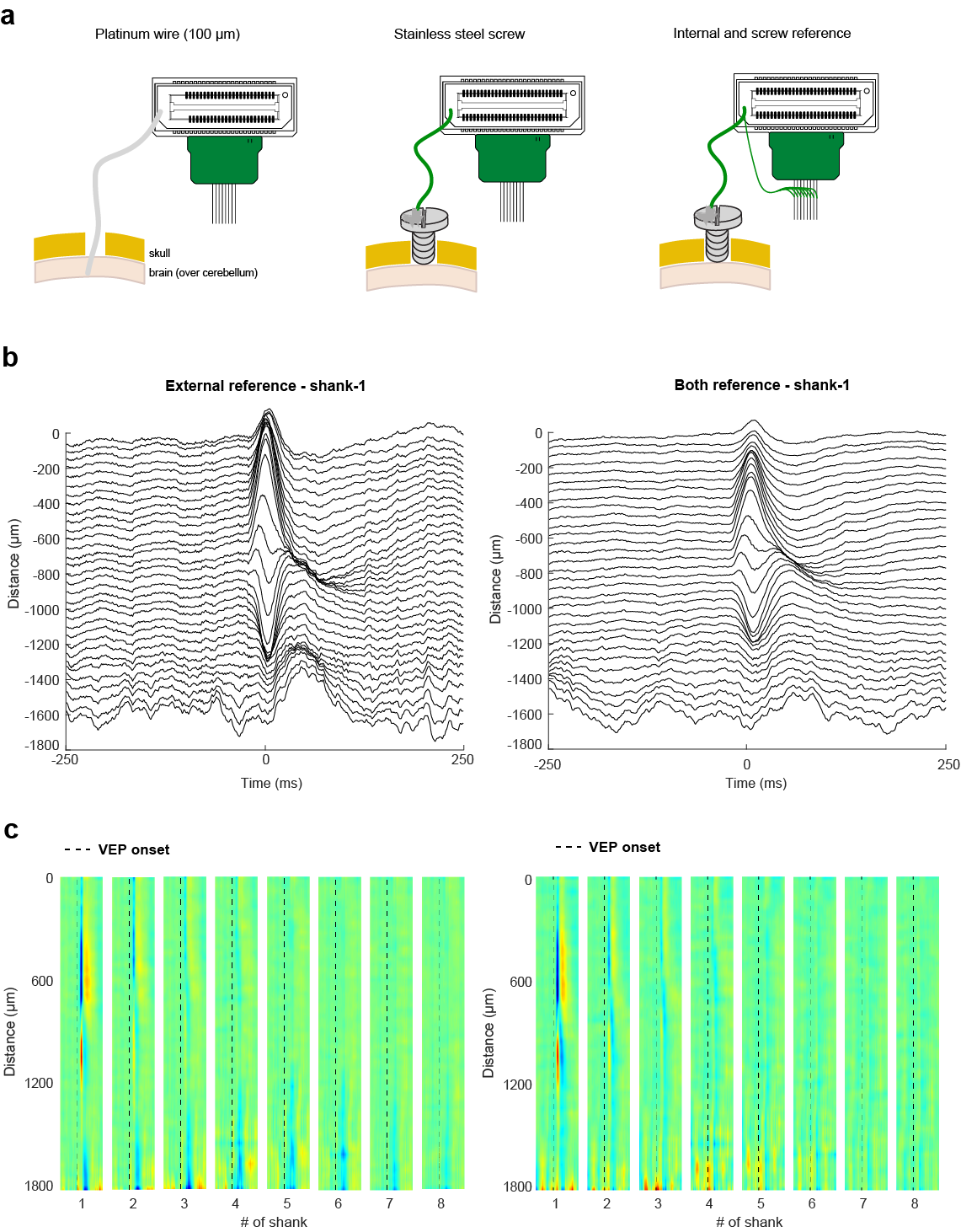
**

**Figure S5. Comparison of reference material and source. (a)** The material of the reference did not change the signal quality measured by the RMS noise and V_refZ value of the SiNAPS system. **(b)** Visual stimuli triggered LFP averages across shank-1 using an external reference (left) or both references (right). To aid visualization, every 4th channel is displayed (zero μm corresponds to the dorsal surface of the brain). As expected, the amplitude of the visual evoked potential was larger when the signal was referenced over the cerebellum only compared to both reference configurations. **(c)** Current source density analysis identified sinks and sources on shanks 1 and 2 in both reference configurations.


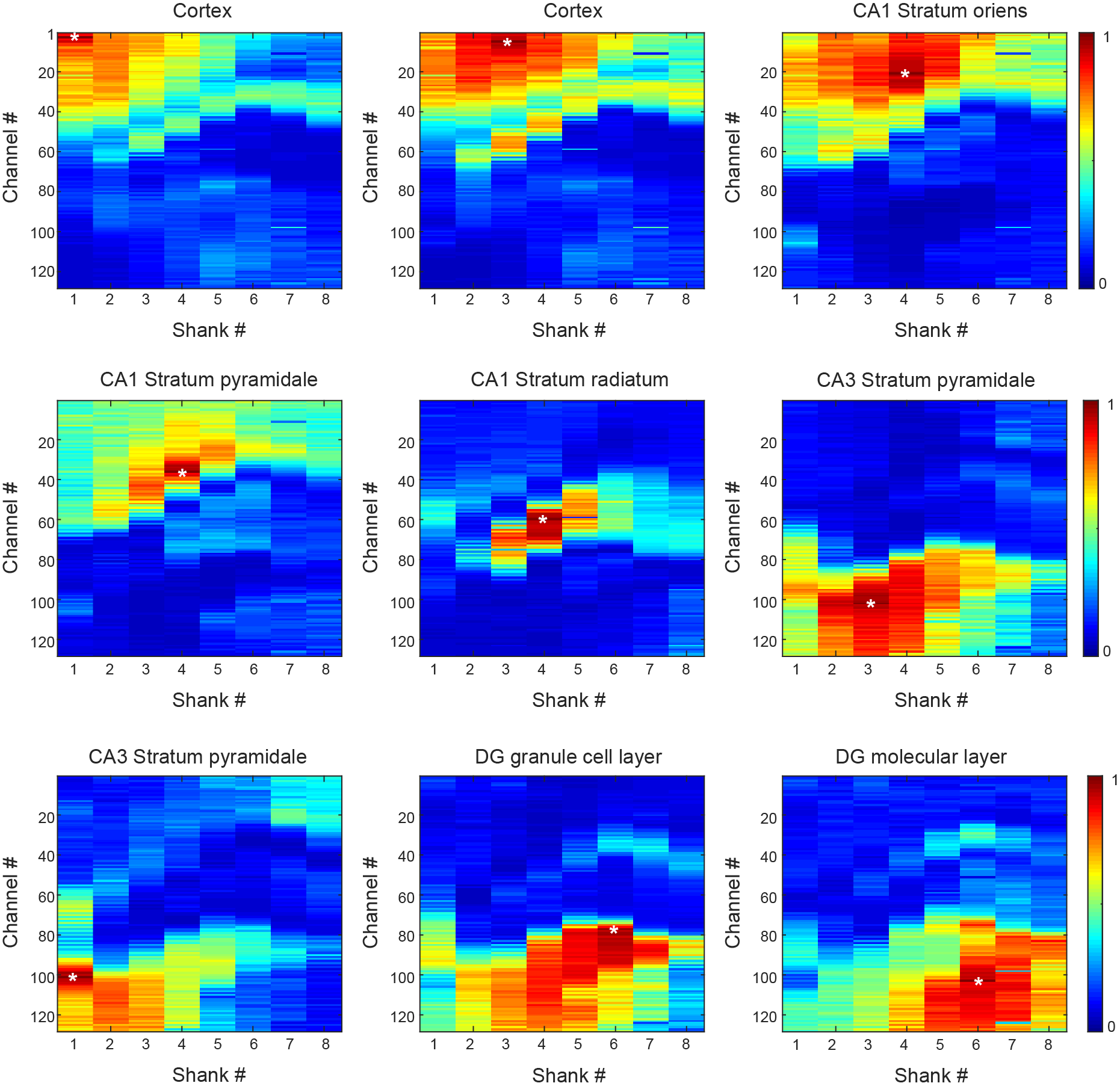


**Figure S6. Coherence maps of gamma activity.** Gamma (30–90 Hz) coherence maps between LFP recorded from 9 reference electrode-pixels (white stars) and all other recording electrode-pixels on a 1024-channel. SiNAPS electrode spanning cortex and the entire dorsal hippocampus in one example session.


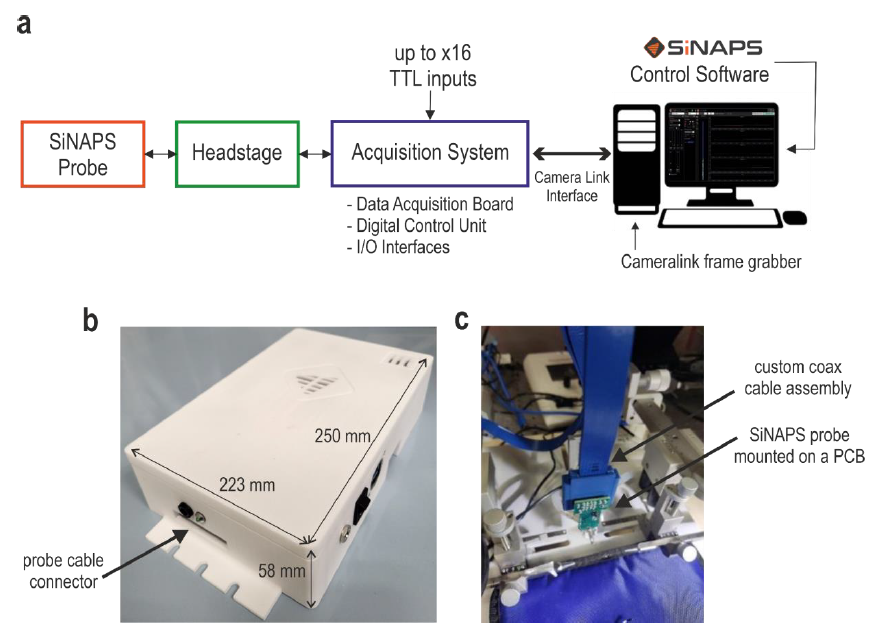


**Figure S7. The SiNAPS Research Platform used in the experiments at LMU.** (**a**) Schematic overview of the system components. (**b**) The Acquisition System includes the data acquisition board, the FPGA-based digital control unit and I/O interfaces for data acquisition, SiNAPS probe control and for synchronously sampling TTL signals from other instruments in the setup and electrophysiological data, both at 20kHz/channel. (**c**) View of a mounted probe and the custom coax cable.


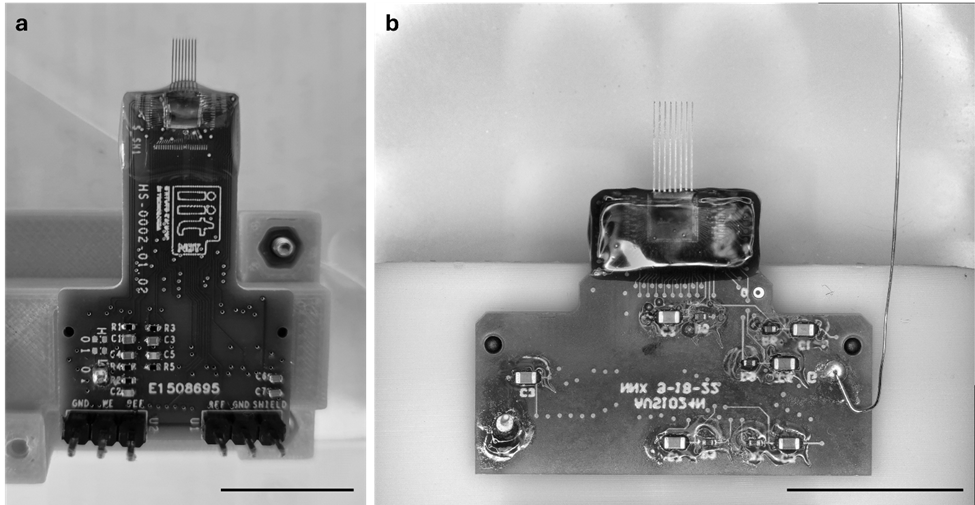


**Figure S8. Optical images of mounted SiNAPS probes.** (a) View of a probe mounted on PCBs for interfacing with the SiNAPS Research Platform. (b) View of a probe mounted by Neuronexus for interfacing with the Smart Box Pro instrument. (Scalebar 1 cm).

| **Animal ID** | **Device ID** | **RMS**  **(μV, mean ± SD)** | **No. non-functional electrode-pixels** |
| --- | --- | --- | --- |
| Mouse_01 | Device - 1 | 6.46 ± 0.54 | 4 |
| Mouse_02 | Device - 1 | 5.83 ± 0.99 | 8 |
| Mouse_03 | Device - 1 | 6.03 ± 0.94 | 10 |
| Mouse_04 | Device - 1 | 4.41 ± 0.48 | 3 |
| Mouse_05 | Device - 2 | 5.17 ± 0.76 | 7 |
| Mouse_06 | Device - 1 | 6.07 ± 0.58 | 18 |
| Mouse_07 | Device_3 | 3.09 ± 0.11 | 18 |

**Table S1**. Summary of electrode noise and yield across experiments. We used three 8-shank SiNAPS probes in 7 animals (one head-fixed acute session / animal). Device-1 was used 5 times and devices-2 and 3 were used once. To determine the noise floor for each recording session, five 100 ms snippets of high-pass filtered (700 Hz) electrophysiology recording data were chosen along the 2-hour session. These snippets did not contain sorted units and did not display motion artifacts. The snippets of data were then joined together in a single 500 ms block which was used to calculate Vrms for each pixel. RMS values in the Table below represent the mean Vrms computed across all operating electrode-pixels.

| **Publication** | **This work** | **Jun et. al., 2017**^2^ | **Steinmetz et. al, 2021**^3^ | **Raducanu et al. 2017**^4^ |
| --- | --- | --- | --- | --- |
| **No. Total Recording Electrode-Channels (NTREC)** | 1024 | 384 | 384 | 1356 |
| **No. shanks** | 4 / 8 | 1 | 1 / 4 | 1 |
| **NTREC / shanks** | 256 / 128 | Up to 384 | Up to 384 | Up to 1356 |
| **Max. inter-electrode distance column / row (μm)** | 28 / 30 | 16 / 20 | 15 / 32 | 22.5 / 22.5 |
| **Shank cross sectional area (μm^2^)** | 4400 | 1680 | 1680 | 5000 |
| **Inter-shank distance** | 300 | NA | 250 | NA |
| **AP noise (uVRMS)** | 6.67 ± 1.02 (300Hz - 5kHz) | 6.36 (300Hz - 10kHz) | 7.74 (300Hz - 10kHz) | for 678 channels: 12.4 (300 Hz–7.5 kHz) |
| **LFP noise (uVRMS)** | 16.45 ± 3.47 (0.1Hz - 300Hz) | 10.32 (0.5Hz - 1kHZ) | 7.78 (0.5Hz - 1kHz) | for 678 channels: 50.2 (1Hz - 1kHz) |
| **Low pass filtering cutoff frequency (Hz)** | f-3dB = 5 kHz | f-3dB = 10 kHz | f-3dB = 10 kHz | f-3dB = 7.5 kHz |
| **CMOS Technology** | 180nm | 130nm | 130nm | 130nm |

**Table S2**. **State-of-the-art of in-vivo validated active dense CMOS probes.** The SiNAPS probes presented in his work provide the largest number of electrode-channels and shanks compared with available CMOS multi-shank devices, thus allowing continuous monitoring of the largest area in the brain.

**Supplementary References:**

1. Claudi, F., Tyson, A.L., Petrucco, L., Margrie, T.W., Portugues, R., and Branco, T. (2021). Visualizing anatomically registered data with brainrender. Elife *10*, e65751. 10.7554/eLife.65751.

2. Jun, J.J., Steinmetz, N.A., Siegle, J.H., Denman, D.J., Bauza, M., Barbarits, B., Lee, A.K., Anastassiou, C.A., Andrei, A., Aydin, Ç., et al. (2017). Fully integrated silicon probes for high-density recording of neural activity. Nature *551*, 232–236. 10.1038/nature24636.

3. Steinmetz, N.A., Aydin, C., Lebedeva, A., Okun, M., Pachitariu, M., Bauza, M., Beau, M., Bhagat, J., Böhm, C., Broux, M., et al. (2021). Neuropixels 2.0: A miniaturized high-density probe for stable, long-term brain recordings. Science (80-. ). *372*. 10.1126/science.abf4588.

4. Raducanu, B.C., Yazicioglu, R.F., Lopez, C.M., Ballini, M., Putzeys, J., Wang, S., Andrei, A., Rochus, V., Welkenhuysen, M., Helleputte, N. van, et al. (2017). Time Multiplexed Active Neural Probe with 1356 Parallel Recording Sites. Sensors *17*, 2388. 10.3390/s17102388.
